# The cost-effectiveness of a treatment-based classification system for low back pain: design of a randomised controlled trial and economic evaluation

**DOI:** 10.1186/1471-2474-11-58

**Published:** 2010-03-26

**Authors:** Adri T Apeldoorn, Raymond W Ostelo, Hans van Helvoirt, Julie M Fritz, Henrika CW de Vet, Maurits W van Tulder

**Affiliations:** 1 Department of Epidemiology and Biostatistics and the EMGO Institute for Health and Care Research, VU University Medical Centre, Amsterdam, the Netherlands; 2 Department of Health Sciences and the EMGO Institute for Health and Care Research, Faculty of Earth and Life Sciences, VU University Amsterdam, the Netherlands; 3 Medical Back Neck Centre, The Hague, the Netherlands; 4 Department of Physical Therapy, University of Utah, Salt Lake City, Utah, and Intermountain Health Care, Salt Lake City, Utah, USA

## Abstract

**Background:**

Systematic reviews have shown that exercise therapy and spinal manipulation are both more effective for low back pain (LBP) than no treatment at all. However, the effects are at best modest. To enhance the clinical outcomes, recommendations are to improve the patient selection process, and to identify relevant subgroups to guide clinical decision-making. One of the systems that has potentials to improve clinical decision-making is a treatment-based classification system that is intended to identify those patients who are most likely to respond to direction-specific exercises, manipulation, or stabilisation exercises.

**Methods/Design:**

The primary aim of this randomised controlled trial will be to assess the effectiveness of a classification-based system. A sample of 150 patients with subacute and chronic LBP who attend a private physical therapy clinic for treatment will be recruited. At baseline, all participants will undergo a standard evaluation by trained research physical therapists and will be classified into one of the following subgroups: direction-specific exercises, manipulation, or stabilisation. The patient will not be informed about the results of the examination. Patients will be randomly assigned to classification-based treatment or usual care according to the Dutch LBP guidelines, and will complete questionnaires at baseline, and 8, 26, and 52 weeks after the start of the treatment. The primary outcomes will be general perceived recovery, functional status, and pain intensity. Alongside this trial, an economic evaluation of cost-effectiveness and cost-utility will be conducted from a societal perspective.

**Discussion:**

The present study will contribute to our knowledge about the effectiveness and cost-effectiveness of classification-based treatment in patients with LBP.

**Trial registration:**

Trial registration number: NTR1176

## Background

Low back pain (LBP) is a common condition. It has a lifetime prevalence of over 70% in industrialised countries, and accounts for considerable healthcare and socioeconomic costs [1,2]. It is often not possible to make a specific diagnosis based on patho-anatomical causes, and 85-95% of the cases are diagnosed as "non specific" LBP [3]. The lack of a clear patho-anatomical basis for LBP has resulted in a large variation in LBP diagnoses, and a multitude of poorly studied interventions [4]. It has been assumed that randomised controlled trials (RCTs) could provide answers to questions such as 'which intervention is most effective for which patient', but after the publication of more than 1,000 RCTs focusing on LBP, there is still a lack of evidence regarding the most effective strategies for matching individual patients to specific interventions. The Cochrane Back Review Group acknowledged the limited role of RCTs in providing useful information on aspects of LBP management other than efficacy and effectiveness, and stated that additional etiological, diagnostic, and prognostic studies are needed to identify varieties of LBP, natural courses, or more homogeneous subgroups of patients with LBP [5]. The identification of homogenous subgroups according to evidence-based classification systems was determined as a priority for primary care research on LBP as early as 1996 [6].

Over the years, many attempts have been made to classify patients with LBP into more homogeneous subgroups, based on the specific interventions that they are likely to respond to. In 2007, Billis et al. identified 39 diagnostic and treatment-based classification systems [7]. Most of the classification systems were based on biomedical patient characteristics, and seldom on psychosocial or biopsychosocial features. The majority of the classification systems was based on a judgemental approach, relying on clinical experience and intuition, and a minority was based on a statistical approach and prospective study designs.

One of the few classification systems that has potentials to improve outcomes is a treatment-based classification approach, originally proposed by Delitto et al. [8]. This approach is based on the patient's history, clinical presentation, and a physical examination. It identifies subgroups of patients who are most likely to respond to the following interventions: (1) direction-specific exercises, (2) manipulation, (3) stabilisation, and (4) traction. Since the early nineties, several derivation and validation studies have focused on these four interventions and the entire classification system (Additional file 1) [9-20]. Two studies have investigated the validity of the entire classification system, but because the studies used different RCT designs, they answered different research questions. Fritz et al. [12] investigated the effectiveness of the overall treatment approach of the classification system (classification decision-making and treatment protocols), whereas Brennan et al. [18] focused on the effectiveness of the classification decision-making. In a RCT, Fritz et al. [12] compared treatment according to the classification system with treatment according to the clinical guidelines (low-stress aerobic exercises and advice to remain active) for 78 patients with acute, work-related LBP. They found statistically significant better results for the outcomes of disability and return to work in patients who were receiving classification-based treatment at the four-week follow-up, but not at the one-year follow-up. Their research addressed the efficacy of the overall treatment approach of the classification system. However, the results may have been influenced by the treatment protocols that were used, and not the classification decision-making process. Brennan et al. [18] classified 123 patients with a duration of LBP of less than 90 days according to the classification system. All patients were randomised to receive direction-specific exercises, manipulation or stabilisation, regardless their classification. Outcomes were compared between patients who had received 'matched treatment' (according to the classification) and 'unmatched' (non-classification) treatment. The authors found a statistically significant reduction in disability, favouring the matched treatment group after 4 and 52 weeks.

The reliability of the classification system has been assessed in four studies. In a study of 43 patients with acute LBP, Fritz et al. [21] investigated the inter-rater reliability of the classification of patients into one of the subgroups by 7 physical therapists (PTs) who were familiar with the system. They achieved a kappa value of 0.49. Kiesel et al. [22] achieved a slightly higher kappa value (0.65) with 8 PTs who were familiar with the classification system and 30 patients with LBP. Heiss et al. [23], in a study in which 45 acute LBP patients were classified by four PTs who were unfamiliar with the classification system, found a low kappa value (0.15). Finally, Fritz et al. [24] investigated the inter-rater reliability of the system in a vignette study with 30 PTs and 123 LBP patients, and reported a kappa value of 0.60. These findings therefore provide preliminary evidence that PTs who are familiar with the classification system can obtain an acceptable level of intra-rater reliability.

It is essential for a treatment-based classification approach to be dynamic, and to incorporate new evidence. Since the original proposal, made by Delitto and colleagues, the criteria for allocating a patient to a specific intervention, and also the intervention procedures, have been revised and updated. However, although this continued evolution of a classification system is necessary in order to incorporate emerging evidence, it does reduce the comparability of studies using different versions of the treatment-based classification approach, and should motivate continued research on the approach.

In summary, the classification system proposed by Delitto and colleagues [8] has evolved considerably, and some promising results have been reported. However, the evidence for its clinical usefulness is still limited. Therefore, the aim of the present study is to evaluate the cost-effectiveness of classification-based treatment, compared to usual care according to national clinical guidelines, in patients with subacute and chronic LBP in primary care in the Netherlands. The present study will focus on a revised version of the Delitto et al. treatment-based classification system [8], based on updated evidence outlined in previous RCTs [25], but is consistent with the concept that sub-groups of patients can be identified, and that the primary subgroups are patients who are likely to respond to direction-specific exercises, stabilisation or manipulation. The aim of this paper is to describe the rationale and design of the study.

## Methods/Design

### Design

**Figure 1:**
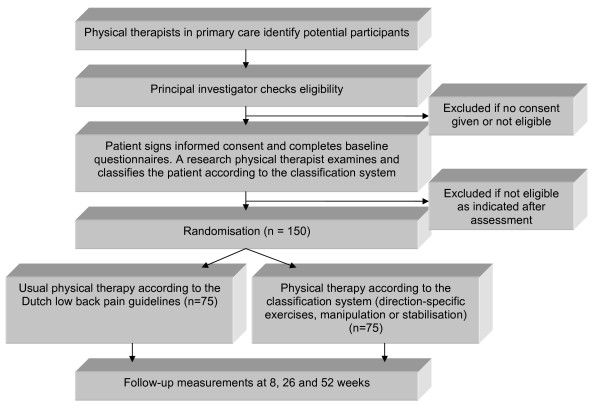
**Design of the study**.

A randomised controlled trial will be conducted, together with a full economic evaluation. Figure 1 provides an overview of the study design.

### Setting

Up to 300 private physical therapy clinics, with more than 1,000 PTs in the city of Amsterdam and the surrounding (rural) area (< 50-kilometre radius), will be invited to participate.

### Ethical approval

In February 2008, the Medical Ethics Committee of the VU University Medical Centre in Amsterdam approved the study protocol (registration number: 2008\5).

### Study population

The study population will consist of patients who seek care in a participating private physical therapy clinic. The inclusion criteria will be: LBP as primary complaint, with or without associated leg pain, age between 18 and 65 years, current episode >6 weeks, able to speak and understand Dutch, and willing to give written informed consent. The exclusion criteria will be: known or suspected specific LBP (e.g. cauda equina compression, fractures), severe radiculopathy, spondylolisthesis (grade 2 or more), serious co-morbidity, serious psychopathology, lumbar spinal surgery in the previous year, more than one low back operation, more than one year of absence from work due to LBP, pregnancy or given birth in the past three months, inability to attend 6 or more regular physical therapy appointments, moderate complaints about one or more items of the Urogenital Distress Inventory (UDI 6, short form) or inability to demonstrate any LBP symptoms during mechanical examination.

### Recruitment of patients

PTs will inform all eligible LBP patients who attend their clinic about the study. Subsequently, the principal investigator (AA) will phone potentially eligible patients to explain the trial procedures in detail and to check the selection criteria. If a patient fulfils the initial requirements for eligibility, an appointment will be made at the private physical therapy clinic that the patient attended, where the patient will be asked to sign an informed consent form and to complete baseline questionnaires. The patient will then undergo a physical examination.

The PTs will also be asked to collect basic data from eligible patients who are not willing to participate, and to record their reasons for declining.

### Research physical therapists

All patients will be examined by one of four research PTs, all of whom have followed a post-graduate course in manipulation and mobilisation techniques, and are certified manual therapists (MTs) in the Netherlands. Two research PTs have a Mechanical Diagnosis and Therapy certificate (MDT or McKenzie method). The two research PTs with no MDT certificate attended a one-day MDT training session supervised by a senior educator of the MDT Institute (HvH) to familiarise them with the MDT system. Prior to the study, the research PTs held meetings to standardise the examination protocol, to train in examination techniques and to examine patients together. The research PTs will examine and classify the patients, but will not be involved in providing the intervention.

### Classification system

**Figure 2:**
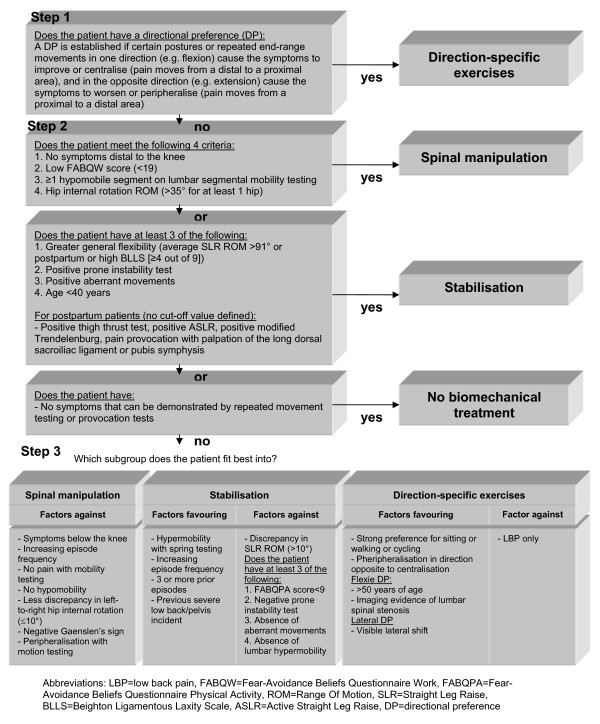
**Modified treatment-based classification system for patients with LBP (>6 weeks) as used in this RCT**.

The classification system that will be used in this study is a revised version of the system used by Brennan et al. [18]. The classification system is a decision-making procedure that is used to allocate patients to one of the following treatment categories: direction-specific exercises, manipulation, or stabilisation (Figure 2).

### Baseline examination

The examination procedure will start with a standardised history-taking, which will include questions on the frequency of episodes, current duration, intensity, and location of the symptoms. Subsequently, a standardised comprehensive physical assessment will be made, together with a neurological examination if indicated. The examination starts with the determination of a directional preference, based on the definition proposed in the MDT classification method (step 1 in Figure 2) [26]. This involves the monitoring of symptomatic and mechanical responses during repetitive end-range lumbar test movements (flexion, extension, side-gliding, or rotation) and/or prolonged positioning. A directional preference is established if certain postures or repeated end-range movements in one direction (e.g. flexion) cause the symptoms to improve or centralise (pain moves from a distal to a proximal area), and in the opposite direction (e.g. extension) cause the symptoms to worsen or peripheralise (pain moves from a proximal to a distal area). Priority is given to peripheral pain locations and their response, regardless of the response of the central pain intensity. The improved or centralised symptoms must be retained in a neutral standing position. A directional preference is not established if the symptoms and the pain immediately reappear. For the sake of clarity, patients with a directional preference do not necessarily have centralisation of the symptoms. After this assessment, several other physical tests, such as the prone instability test, thigh thrust test, and the active straight leg raise test will be performed (step 2 in Figure 2). The operational definitions of these tests are summarised in the Appendix (Additional file 2). The classification decision-making has a hierarchical structure, however, all patients will undergo the entire examination procedure. In cases of uncertainty about the primary classification, extra criteria, such as three or more prior LBP episodes in the medical history and a visible lateral shift during the examination, will be used to determine the patient's subgroup (step 3 in Figure 2). Directly after this examination the research PT will determine the patient's classification and the level of firmness of the conclusion: level 1. there is an obvious primary classification; level 2. two of the three classifications are plausible; and level 3. none or all of the three classifications are plausible. The patient will not be informed about the results of the examination. Reasons for exclusion, such as inability to demonstrate any LBP symptoms during the examination, will be registered. It is the intention that the principal investigator (AA) will examine all patients. To identify patients reliably, one of the three other research PTs, depending on availability, will attend the examination procedure. When two research PTs examine a patient, the primary classification will be reached by consensus.

### Treatment allocation

To conceal the treatment allocation, an independent researcher who is not involved in the selection of the patients will randomise each patient to one of the two treatment groups. Randomisation lists will be generated by computer before the study starts, pre-stratified for the duration of the complaints (6-12 weeks or >12 weeks) and disability (Oswestry Disability Index [ODI] <25% or ≥25%) to prevent unequal distribution. To prevent unequal treatment-group sizes, the patients will be randomised according to a stratified block randomisation method, in blocks of four. No treatment will be given prior to randomisation.

Directly after the randomisation procedure, the PT who will provide treatment will be informed about the group allocation. Patients will be scheduled for their first treatment session as soon as possible in the private practice they attended.

### Physical therapists in the participating clinics

All patients will be treated by PTs in the participating clinics. In each clinic, at least one PT will be instructed about the classification system, and at least one PT will be instructed about the usual care procedure, depending on preference and expertise. PTs who will provide manipulation treatment must be licensed, post-graduate MTs or in the 4^th ^and final year of the MT master education program at the Educational Center for Musculoskeletal Therapies (SOMT) in the Netherlands. No extra qualifications will be required to treat patients according to the classifications of direction-specific exercises and stabilisation. The number of years of experience of the PTs, and their post-graduate education in MT and/or MDT, will be recorded for both treatment groups. To minimise contamination, the PTs who will provide the classification-based treatment will not be allowed to provide usual care, and vice versa.

The PTs who will provide the classification-based treatment will follow a five-hour training session in which the treatment protocols will be described and discussed. Experts on direction-specific exercises (HvH) and stabilisation will provide the training. The PTs will receive a detailed manual of the study, and a translation of the registration forms and treatment protocols used in the Brennan et al. study [18]. The PTs providing usual care will receive a two-hour training course in the rationale and treatment components described in the LBP guidelines issued by the Royal Dutch College for Physical Therapy (KNGF) [27,28].

### Intervention according to the Dutch LBP guidelines

Patients assigned to usual care will receive PT or MT according to the current Dutch LBP guidelines [27,28]. The PTs providing usual care will not be informed about the physical examination and classification procedure, and will therefore reassess the patient to obtain the relevant information for treatment. However, to avoid inconvenience for the patient, and to increase the chance that the patient remains unaware of the treatment allocation, the PT will receive a written description of the patient's medical history from the research PTs. The patients will receive supervised, individually tailored treatment based on their clinical presentation. Treatment techniques vary within and among PTs, and depend on the preferences and expertise of the PTs. The focus of the treatment will be on improving function and participation. Interventions that are commonly used in Dutch primary care are 'hands-on' muscular mobilisation techniques, specific articular mobilisation and manipulation techniques, strength and stability exercises, massage, and cognitive, respondent and operant techniques, such as relaxation exercises and graded activity programmes. Decisions about the frequency and the number of sessions are left to the discretion of the PT. The number of treatment sessions, the treatment modalities, the treatment goals, any deviations from the protocol, and reasons for prematurely terminating treatment, will be recorded.

### Interventions according to the classification system

Before providing any treatment, the PTs in this group will be informed about the results of the classification procedure. Patients will receive either direction-specific exercises, spinal manipulation, or stabilisation.

#### I. Direction-specific exercises

Patients will perform exercises in the direction that matches their directional preference: extension, flexion, lateral (side-gliding right or left) or flexion-rotation (right or left). For each direction is a treatment protocol which contains patient-generated forces (sustained positions and/or repeated movements) and PT-generated forces (mobilisations in the direction of preference). The protocol can be adapted to the patient's pain responses. All patients will receive a booklet containing information about directional preference and centralisation, the exercises, and the correct performance and frequency of each exercise. The patients will be instructed to perform the exercises four times a day, to correct their posture in the preferred direction, and to avoid activities and positions that increase the intensity or peripheralisation of symptoms. The patients will also be asked to keep an exercise logbook to monitor their compliance.

#### II. Spinal manipulation

Patients will be treated with high velocity thrusts directed at the spinal levels from T12 to L5 and/or the sacroiliac region. Decisions about the choice of the technique (long lever to manipulate multiple levels or short lever to manipulate one specific level) and the location of the forces are left to the discretion of the PTs. The most common spinal manipulation technique in the Netherlands is one of the two technique options in the Brennan et al. study [18], with the patient lying on his/her side. The symptomatic side is manipulated first. If the patient cannot specify a symptomatic side, the PT can select a side. During each session, the PT can make a maximum of four attempts (two on each side) to achieve a cavitation (i.e. 'a pop') heard or felt by the PT or the patient. Each session will end with 10 active flexion and extension movements of the lumbar spine in a supine or quadruped position within the limits of pain. The choice of this flexion-extension exercise will be made by the PT. Patients will be instructed to perform this active exercise once a day. The PT will record the number of attempts, whether or not a cavitation is heard or felt, and the technique that is used (long or short lever). The PT is allowed to provide spinal manipulation treatment for a maximum of 6 sessions.

#### III. Stabilisation

First, the patients will be taught to activate their local stability system to control neutral joint position in a supine position [29]. This (slight) co-activation of the lumbar multifidus with the transversus abdominus ('drawing in' the stomach) must be accompanied by minimal trunk movements and relaxation of global muscles such as the upper abdominal muscles. The patients will then be instructed to perform these abdominal bracing exercises ('drawing in' the stomach with normal breathing) during strengthening exercises in standing, quadruped, and side-support positions. Apart from these standardised exercises, activity of the local and global muscle systems will be trained in positions and movements that aggravate the patient's pain, in order to restore functional capacity and to improve dynamic control. The patients will also receive an instruction booklet, and will be instructed to perform the exercises once a day and to keep self-monitoring exercise diaries.

For all classification-based interventions, the treatment frequency, amount and type of exercises, and the duration and progression of the programme will be recorded, and directed by the judgment of the PT. The treatment frequency will only be restricted for spinal manipulation (maximum of 6 sessions). The patients will be treated according to their primary classification for a minimum of 4 weeks, during which the PT will be allowed to change the treatment strategy, if (1) the functionality of the patient improves significantly (a reduction of >30% on the ODI, compared to the score at baseline) or (2) the symptoms are severely aggravated. After the first 4 weeks, the PT will be allowed to provide follow-up interventions. At the end of each patient's treatment period, the PT will note the degree of satisfaction with the classification-based treatment, measured on a 5-point Likert scale ranging from very satisfied to very dissatisfied.

In both treatment groups, the patients are requested not to receive any co-interventions apart from the physical therapy (such as complementary and/or alternative medicine [CAM]) during the intervention period. All patients will be asked to stay active, and to engage in general exercise activities (e.g. a sports club). The principal investigator will regularly monitor the PTs for compliance with the treatment protocols, by means of visits, phone calls and e-mail contact.

### Blinding

The PTs will be asked not to disclose the treatment allocation to the patients, and the success of the blinding will be evaluated at the 8-week follow-up. The research assistants and the participating statistician will be blinded for the patient's group assignment. The research PTs and the treating PTs cannot be blinded for the treatment allocation, but they will not be involved in the outcome date-collection.

### Measurements

The baseline questionnaire will include questions about socio-demographic characteristics (e.g. age, gender, marital status, etc.) and all primary and secondary outcome measures.

### Primary outcome measures

Global perceived recovery will be measured by self-assessment on a 7-point Likert scale ranging from "completely recovered" to "worse than ever" [30]. This will be dichotomised into success (complete and much recovered) and non-success (slightly recovered, no change, slightly worse, much worse and worse than ever). This measure has been found to be useful in LBP research [31].

Functional status will be measured according to the Dutch translation of the ODI, version 2.1a [32,33]. The ODI is sub-divided into 10 sections related to activities of daily living. Each section consists of 6 graded responses, scored 0-5, and the total score can range from 0 (no difficulty at all) to 50 (maximal difficulty). The ODI is widely used in LBP studies, and has also been used as a primary outcome measure in studies focusing on the treatment-based classification approach [12,18].

Pain intensity over the previous week will be measured on an 11-point numerical rating scale (0 = no pain to 10 = worst imaginable pain), with reliability, validity, and sensibility to change that are commonly accepted in LBP research [34-36].

### Secondary outcome measures

General health status will be evaluated with the Short Form 36 (SF-36) [37]. It consists of 36 questions that can be aggregated to form eight sub-scales (physical functioning, mental health, general health perceptions, pain, role limitations physical, role limitations emotional, social functioning, and vitality) and two sum-scales (physical and mental component scales). The scores on all sub-scales range from 0-100, with higher scores indicating better outcomes. It is a widely used measurement instrument with satisfactory validity, reproducibility and responsiveness to change [38], and the Dutch translation has been found to be sufficiently valid [39].

Health-related quality of life will be measured with the EuroQol (EQ-5D) [40]. This questionnaire assesses 5 dimensions (mobility, self-care, usual activities, pain/discomfort and anxiety/depression) on a 3-point scale; no problems, moderate problems and severe problems. The questionnaire is appropriate for estimating quality-adjusted life-years (QALYs), and can be used for cost-utility analysis. The total score is expressed in utilities according to the Dolan model [41]. The questionnaire has been found to have adequate psychometric properties, and is commonly used in LBP research [42]. QALYs will be calculated by multiplying the utility of a health state by the time spent in this health state, based on the Dutch valuation tariff [43].

Fear-avoidance beliefs will be measured with the Dutch version of the Fear-Avoidance Beliefs Questionnaire (FABQ) [44]. It consists of two multi-item scales, measuring the patient's beliefs about how physical activity (FABQPA, 5 questions) and work (FABQW, 11 questions) affect their LBP. Following the recommendations of Waddell et al. [44], only 4 items will be included to score the FABQPA sub-scale (range 0-24) and 7 items to score the FABQW sub-scale (range 0-42). Higher scores indicate more fear-avoidance beliefs.

Psychosocial status will be measured with the Örebro Musculoskeletal Pain Screening Questionnaire (ÖMPSQ) [45]. This questionnaire contains 25 items, divided into five groups (function, pain, psychological factors, fear-avoidance, and miscellaneous), 21 of which are scored on a 0-10 scale. The scores of the items are summed to provide a total score range of 0-210, with higher scores indicating a higher risk of a poor outcome. The ÖMSPQ has been found to be a reliable and valid predictor of outcome for patients with acute, subacute and chronic LBP [46,47].

Work absenteeism and presenteeism will be measured with the Productivity and Disease Questionnaire (PRODISQ) [48]. This questionnaire was developed and validated in samples of patients and workers in the Netherlands, and covers all relevant aspects of the relationship between health and productivity.

Direct and indirect costs will be measured by means of self-completed cost diaries [49]. The following costs will be evaluated: i) health care costs (e.g. primary care, medical specialist care, prescription of medication, professional home care and hospitalisation); ii) patient and family costs (out-of-pocket expenses, such as over-the-counter medication and the costs of paid and unpaid help); and iii) costs of loss of productivity (work absenteeism and presenteeism, and costs due to days of inactivity for patients without a paid job).

Table 1 presents an overview of the data-collection procedures.

**Table 1:** Overview of the data-collection

Instrument	Baseline	8 weeks	26 weeks	39 weeks	52 weeks
Demographic data	X				
Physical characteristics and primary classification	X				
					
*Primary outcome*					
Global perceived recovery		X	X		X
Disability (ODI)	X	X	X		X
Pain intensity (NRS)	X	X	X		X
					
*Secondary outcome*					
General health (SF-36)	X	X	X		X
Health-related quality of life (EQ-5D)	X	X	X		X
Fear-avoidance beliefs (FABQ)	X				X
Psychosocial status (ÖMPSQ)	X				X
					
*Economic evaluation*					
Costs (PRODISQ and cost diary)		X	X	X	X

Abbreviations: ODI = Oswestry Disability Index, NRS = Numerical Rating Scale, SF-36 = Short Form 36, EQ-5D = EuroQol, FABQ = Fear-Avoidance Beliefs Questionnaire, ÖMPSQ = Örebro Musculoskeletal Pain Screening Questionnaire, PRODISQ = PROductivity and DISease Questionnaire

### Sample size

Power calculations, based on the studies carried out by Brennan et al. [18] and Fritz et al. [12], were performed for the 3 main outcomes (for all: power 0.9; alpha 0.05). To detect a clinically relevant mean difference between the classification group and the usual care group of 9 points on the ODI (standard deviation [SD] 16), 55 patients are needed per group. To detect a clinically relevant mean difference of 2 points (SD 2) for pain (11-point numerical rating scale), 2 groups of 21 patients are needed. To detect a 20% difference in the dichotomised global perceived recovery (recovered vs. not recovered), 68 patients are needed per group. Anticipating a potential drop-out of 10%, 75 participants per treatment group (total n = 150) will be recruited.

### Data-analysis

Baseline characteristics of the two intervention groups with regard to the most important prognostic indicators and main outcome measures will be compared to assess the adequacy of the randomisation. If necessary, adjustments will be made for baseline characteristics. The primary analysis will be an intention-to-treat analysis. A per protocol analysis will be performed to estimate the extent to which protocol deviations might have biased the results. The data will be analysed in a linear mixed model, with responses at 0 (baseline), 8, 26 and 52 weeks. In this model, the effect of interest is the time × treatment interaction. Estimates of treatment effects, with 95% confidence intervals between baseline and follow-up measurements, will be calculated and compared between the two treatment groups. Multivariable regression analysis will be used to adjust for possible baseline differences, and multilevel analysis will be performed, with PT, patient, and time of measurement as levels. Additionally, as a secondary analysis, a responder analysis will be performed according to the cut-off values as proposed by a consensus panel [50]. If there are many missing data, the missing data will be imputed with multiple imputation techniques.

### Economic evaluation from a societal perspective

The cost-effectiveness analysis will be carried out from a societal perspective. The total back-pain-related costs for patients in both intervention groups will be compared with the health effects of the two groups, and cost-effectiveness ratios will be calculated for the primary outcome measures. The economic evaluation will be performed according to the intention-to-treat principle. Costs will be valued according to the guidelines published in the updated handbook for economic evaluation in the Netherlands [51]. The costs of production losses due to LBP will be estimated for both paid and unpaid labour [52]. For paid labour, the costs will be calculated, using both the human capital approach and the friction cost approach. For unpaid labour, the indirect costs will be estimated as the costs of production losses due to LBP, e.g. voluntary work and household work, based on shadow prices. The costs will be summed for each individual patient, and bootstrapping will be used to make a pair-wise comparison of the mean differences in total costs between the two groups. Confidence intervals will be obtained by bias-corrected and accelerated bootstrapping, using 2000 replications [53].

Cost-utility and cost-effectiveness ratios will be estimated, using bootstrapping techniques. The differences in costs will be compared, and related to the differences in effects (global perceived recovery, functional status and pain intensity) and differences in QALYs. Bootstrapped cost-effect pairs will be used to estimate cost-effectiveness planes and cost-effectiveness acceptability curves. Cost-effectiveness acceptability curves indicate the probability that a treatment is cost-effective at a specific ceiling ratio, which is the amount of money society is willing to pay to gain one extra unit of effect. Sensitivity analysis will be performed on the most important cost-drivers, in order to assess the robustness of the results.

## Discussion

There is an increasing interest in classification systems for LBP patients that can be used to guide treatment. One of the classification systems that has potentials to improve the treatment decision-making process and clinical outcomes is a classification system that was originally proposed by Delitto et al. [8]. To critically examine and test the strength of this system, we designed an RCT, together with an economic evaluation to investigate whether treatment according to this system is more cost-effective than usual care in reducing pain and improving functioning in patients with subacute and chronic LBP in primary care.

### Contrast between interventions

In both intervention groups personalised, individually tailored sessions will be given, and some of the same examination and treatment techniques will be used. However, some important features will account for the contrast between the two groups. Patients in the classification-based treatment group will be classified according to a standardised examination protocol, and will be treated according to standardised treatment protocols. In the usual care group however, the examination techniques are expected to vary greatly among the PTs [54], and the same examination findings can lead to a wide range of treatment goals and strategies.

### Modifications

The classification system is not a static system, and some modifications have been made, based on new evidence [25], and based on the context in which physical therapy is provided within the Dutch health care system.

Patients with moderate complaints about one or more items of the Urogenital Distress Inventory (UDI 6, short form) will be excluded from the study. There are indications that patients with a combination of LBP and continence disorders require tailored treatment that is partially beyond the scope of the classification system [55]. The UDI 6 contains 6 questions on symptoms of irritation, stress, and obstruction/discomfort [56]. Patients can indicate whether a symptom is present, and if so, the degree to which it bothers them (not at all, slightly, moderately or greatly). The UDI 6 is a widely used, scientifically feasible questionnaire for urinary incontinence evaluation purposes, and has been translated and validated in Dutch [57].

Previous studies, carried out by Fritz et al. [12] and Brennan et al. [18], required that patients had at least a moderate level of disability due to LBP. Fritz et al. [12] required that patients had work restrictions, while Brennan et al. [18] required that patients had an ODI score >25%. In the present study, the level of disability is not an inclusion criterion, and therefore the participants may have less disability due to LBP than the participants in previous studies.

In the original approach developed by Delitto et al. [8], and in updated versions used by Fritz et al. [12] and Brennan et al. [18], centralisation was considered as the primary criterion for the classification of direction-specific exercises. In the present study however, directional preference has been recognised and implemented as a key examination finding for this classification [15,25]. Directional preference is a broader construct than centralisation, and it is therefore expected that in the present study more patients will be classified for direction-specific exercises than in previous studies, in which centralisation was the primary examination criterion [12,18].

The criteria for manipulation classification have been revised, based on the studies carried out by Flynn et al. [11] and Childs et al. [14], and in the present study four of these five criteria that they identified will be applied. The criterion of a recent onset of symptoms (<16 days) will be omitted in the present study, since the Dutch guidelines for general practitioners [58] and the multidisciplinary guidelines [59] discourage PT in the acute phase of LBP.

Patients who will be treated according to the manipulation treatment protocol, will be treated with manipulation techniques only, because recent research suggests that these patients may only be responsive to manipulation [11,14,60]. In previous studies, patients in this subgroup could also be treated with mobilisation techniques [12,18]. There is no current evidence for the supremacy of one single manipulation technique [25], so the choice of the manipulation technique will be left to the discretion of the PTs.

No patients in the present study will be classified in the traction subgroup. Although this subgroup is likely to be quite small [19], the exclusion of this treatment may influence the effectiveness of the approach.

Finally, the classification system, which was originally developed for patients with acute low back pain [8], has undergone some validation studies for patients with subacute and chronic LBP [14,15,18,20]. Researchers have suggested that it is more appropriate to subgroup and treat patients with chronic LBP according to psychosocial symptoms instead of the biomechanical signs and symptoms used in the classification system [61,62]. However, although it is well recognised that some key psychosocial factors are overall predictors of outcome [63], there is limited and controversial evidence that these factors can indicate a specific type of treatment [64]. This study will make it possible to investigate the usefulness of the classification system for patients with more chronic LBP symptoms.

The results of this trial will be available in November 2010.

## Competing interests

The authors declare that they have no competing interests.

## Authors' contributions

AA was responsible for developing, translating and implementing the physical examination and treatment protocols, and the preparation of the manuscript. RWO, MWvT and HCWdV were responsible for the study design, the acquisition of funding for the study and the supervising of the protocols. HvH participated in establishing the physical examination and treatment protocols, and instructed the participating physical therapists in direction-specific exercises. JMF contributed to the design of the study, and provided the treatment protocols. All authors have read, revised and approved the final manuscript.

## Pre-publication history

The pre-publication history for this paper can be accessed here:

http://www.biomedcentral.com/1471-2474/11/58/prepub

## Supplementary Material

Additional file 1 **Table S1.Treatment-based classification studies**. It contains relevant publications of the treatment-based classification system that we are investigating. Click here for file

Additional file 2 **Operational definitions for the physical measurements (= Appendix)**. It contains the tests that we will use to classify the patients. Click here for file
