# Supplementary material for: The cost-effectiveness of a treatment-based classification system for low back pain: design of a randomised controlled trial and economic evaluation
Source: BMC Musculoskelet Disord. 2010 Mar 26;11:58. doi: 10.1186/1471-2474-11-58 (PMC2859390; doi:10.1186/1471-2474-11-58)
Supplement: Additional file 1 — Table S1.Treatment-based classification studies. It contains relevant publications of the treatment-based classification system that we are investigating. [file 1471-2474-11-58-S1.DOC]

| **Classification** | **Derivation** | **Study design** | **Number of patients** | **Duration of LBP** | **Follow-up time** | **Validation** | **Study design** | **Number of patients** | **Duration of LBP** | **Follow-up time** |
| --- | --- | --- | --- | --- | --- | --- | --- | --- | --- | --- |
| - **Direction-specific exercises** | McKenzie & May [9] | - | - | A, SA, C | - | Long et al. [15] | RCT | 230 | A, SA, C | 2 weeks |
|  | Delitto et al. [10] | RCT | 24 | A | 5 days | Browder et al. [20] | RCT | 48 | A, SA, C | 1, 4 and 26 weeks |
|  | Schenk et al. [13] | RCT | 25 | A, SA (?) | time between the 1^st^ and 3^rd^ intervention |  |  |  |  |  |
| - **Manipulation** | Flynn et al. [11] | pros. cohort | 71 | A, SA, C | 1 week | Childs et al. [14] | RCT | 131 | A, SA, C | 1, 4 and 26 weeks |
| - **Stabilisation** | Hicks et al. [16] | pros. cohort | 54 | A, SA, C | 8 weeks |  |  |  |  |  |
|  | Fritz et al. [17] | cross-sectional | 49 | A, SA, C | - |  |  |  |  |  |
| - **Traction** | Fritz et al. [19] | RCT | 64 | A, SA, C | 2 and 6 weeks |  |  |  |  |  |
| - **Treatment-based classification system** |  |  |  |  |  | Fritz et al. [12] | RCT | 78 | A | 4 and 52 weeks |
|  |  |  |  |  |  | Brennan et al. [18] | RCT | 123 | A, SA | 4 and 52 weeks |

Abbreviations: LBP=low back pain, A=acute, SA=subacute, C=chronic, RCT=randomised controlled trial, pros=prospective
