# Supplementary material for: The cost-effectiveness of a treatment-based classification system for low back pain: design of a randomised controlled trial and economic evaluation
Source: BMC Musculoskelet Disord. 2010 Mar 26;11:58. doi: 10.1186/1471-2474-11-58 (PMC2859390; doi:10.1186/1471-2474-11-58)
Supplement: Additional file 2 — Operational definitions for the physical measurements (= Appendix). It contains the tests that we will use to classify the patients. [file 1471-2474-11-58-S2.DOC]

**Patient standing**

| **Test** | **Procedure and criteria** |
| --- | --- |
| - Lateral shift (list) | Visible asymmetrical alignment of the spine in the frontal plane to the right or left. |
| - Flexion, extension and lateral flexion mobility of the lumbar spine | The patient is asked to flex, extend and sidebend as far as possible. The examiner assesses the mobility as hypermobile, normal, or hypomobile. |
| - Aberrant movements | The patient is asked to flex the trunk forward as far as possible and bend back to the erect position. The existence of any of the following five abnormalities is noted. The score is positive if one or more aberrant movements are present. |
| 1. Painful arc in flexion | Transient mid-range pain felt during full flexion. |
| 2. Painful arc on return | Transient mid-range pain felt on the way up from full trunk flexion. |
| 3. Gower sign (‘thigh climbing’) | Using the hands for assistance during return from the flexed to the erect position. |
| 4. Instability catch | Any sudden acceleration, deceleration or lateral deviation of the trunk during full flexion or from the flexed to the erect position. |
| 5. Reversal of lumbopelvic rhythm | The patient bends the knees and shifts the pelvis anteriorly before returning to erect position. |
| - Modified Trendelenburg test | The patient is instructed to raise one leg at 90° hip flexion. The test is positive if the pelvis is descending on the flexed side. |
| - General ligamentous mobility on the Beighton Ligamentous Laxity Scale (BLLS) | The test consists of four bilateral tests: passive hyperextension of the elbow greater than 10°; passive hyperextension of the fifth finger greater than 90°; passive abduction of the thumb to contact the forearm; and passive hyperextension of the knees greater than 10°. The last test consists of placing both hands flat on the floor while bending forward without flexing the knees. A point is given for each test the subject can perform. The scores range from 0 to 9. With a test result of ≥4, hypermobility is generally considered to be present. |

**Patient lying supine**

| - Straight leg raise range of motion | The examiner raises the patient’s leg passively at the ankle with one hand, while the other hand maintains the knee in extension. The leg is raised slowly to the maximum tolerated (not until the onset of pain) straight leg raise. Mobility is recorded. |
| --- | --- |
| - Pain provocation test of the pubis symphysis | The examiner gently palpates the front side of the pubic symphysis. The test is positive if the pain persists for at least 5 seconds after the examiner’s hand is removed. |
| - Pelvic torsion or Gaenslen’s test | One leg is bent in full hip and knee flexion and the other extended with the shank over the edge of the table. The examiner simultaneously applies over-pressure to the flexed knee in a superior/posterior direction, and a downward force on the extended leg. The test is positive if the patient experiences familiar pain in the sacroiliac region. The test is performed on both sides. |
| - Thigh thrust or posterior shear test | The examiner flexes the patient’s hip on the painful side to 90°. The examiner places one hand under the sacrum and applies a downward (posterior) force through the line of the femur with the other hand. The test is positive if the patient experiences their familiar pain in the sacroiliac region. |
| - Active straight leg raise test | The patient lies with legs straight and feet 20cm apart, and is instructed to raise one leg 5cm above the couch without bending the knee. The test is positive if the patient notices (slight or severe) heaviness in one or both legs. |

**Patient lying prone**

| - Posteroanterior tests for pain provocation and mobility | Segmental mobility testing of the lumbar spine is performed by the examiner placing the hypothenar eminence of the testing hand over the spinous process of the segment to be tested. This hand is reinforced with the other hand. The elbows and wrists are extended. The examiner gradually moves the body weight forwards and applies a gentle, but firm, anteriorly directed pressure on the spinous process. The examiner assesses the mobility of the five different lumbar levels as hypermobile, normal, or hypomobile, and notes whether pain is provoked. |
| --- | --- |
| - Hip internal rotation range of motion | The patient lies in a prone position with legs straight and feet 20cm apart. The examiner, supporting the patient's ankle on the side being examined, bends the patient’s knee to 90° and passively rotates the hip internally until the pelvis starts to move. Mobility is recorded with a goniometer which is placed on the outside of the patient's ankle on the side being measured. |
| - Test of the long dorsal sacroiliac ligament | The examiner palpates the areas above both of the patient’s sacroiliac joints. The test is positive if at least on one side the pain persists for a minimum of 5 seconds after the examiner’s hand is removed. |

**Patient lying** half-supine

| - Prone instability test | The patient lies prone on the examination table, with the legs over the edge of the table and the feet resting on the floor. While the patient rests in this position, the examiner applies a posteroanterior pressure on the lumbar spine to identify a painful segment as previously described. Any provocation of pain is reported. The patient then lifts the legs off the floor to a height of 10-20cm with straight knees, while holding the table to maintain position. In this position, passive intervertebral motion testing is applied again to the segments that were identified as painful. The test is positive if pain is present in the first position but subsides in the second position. |
| --- | --- |
